# Devising Mobile Sensing and Actuation Infrastructure with Drones

**DOI:** 10.3390/s18020624

**Published:** 2018-02-19

**Authors:** Mungyu Bae, Seungho Yoo, Jongtack Jung, Seongjoon Park, Kangho Kim, Joon Yeop Lee, Hwangnam Kim

**Affiliations:** School of Electrical Engineering, Korea University, Seoul 02841, Korea; nardyen@korea.ac.kr (M.B.); pen0423@korea.ac.kr (S.Y.); skylover89@korea.ac.kr (J.J.); psj900918@korea.ac.kr (S.P.); mybalam2k@korea.ac.kr (K.K.); charon7@korea.ac.kr (J.Y.L.)

**Keywords:** wireless sensor and actuator networks, drone network, connectivity, architectures and applications for the Internet of Things, opportunistic and delay-tolerant networks

## Abstract

Vast applications and services have been enabled as the number of mobile or sensing devices with communication capabilities has grown. However, managing the devices, integrating networks or combining services across different networks has become a new problem since each network is not directly connected via back-end core networks or servers. The issue is and has been discussed especially in wireless sensor and actuator networks (WSAN). In such systems, sensors and actuators are tightly coupled, so when an independent WSAN needs to collaborate with other networks, it is difficult to adequately combine them into an integrated infrastructure. In this paper, we propose drone-as-a-gateway (DaaG), which uses drones as mobile gateways to interconnect isolated networks or combine independent services. Our system contains features that focus on the service being provided in the order of importance, different from an adaptive simple mobile sink system or delay-tolerant system. Our simulation results have shown that the proposed system is able to activate actuators in the order of importance of the service, which uses separate sensors’ data, and it consumes almost the same time in comparison with other path-planning algorithms. Moreover, we have implemented DaaG and presented results in a field test to show that it can enable large-scale on-demand deployment of sensing and actuation infrastructure or the Internet of Things (IoT).

## 1. Introduction

Wireless sensor and actuator networks (WSAN) [1] consist of sensors and actuators that are usually supposed to have at least one wireless communication module to communicate with the sink node. The administrator can control the system by collecting the data from sensors, analyzing the data to generate actuation commands and sending them to the actuators. However, it is hardly feasible to combine independent and isolated WSANs into a new additional service network since sensors are tightly coupled with actuators. This tight coupling may result in the following: the actuation should correspond to the sensor data, and the actuator can affect the sensor data after the actuation, so that the actuator and the sensor may have a high spatial correlation. Furthermore, tight coupling is inevitable in traditional WSAN because sensing devices cannot freely create a network of heterogeneous devices due to their limited capabilities of communication and processing. Even if the technical aspect is excluded, many vendors operate both sensors and actuators together since they require appropriate sensing data for their service.

On the other hand, a novel network paradigm, called the Internet of Things (IoT), has emerged recently. The paradigm should facilitate the communication of various existing networks with each other to collect data or deploy new services, so that general users are able to access any network with their mobile devices much more easily. According to the Ericsson mobility report [2], the number of non-cellular-based machine-to-machine (M2M) devices will dramatically increase from 6.4 billion–17.4 billion from 2017–2023. Moreover, [3] notes that the IoT will be powered by a trillion sensors in 2020, and the IoT could provide an economic impact of 14 trillion by 2020. Following these statistical reports, WSAN could be one of the core infrastructures to implement the IoT.

Additionally, new services in the paradigm demand separation between the sensor network and the actuator network [4,5,6,7], which means that they decide the requirements for the service-related actuator or run it with sensing data obtained from different sensor networks. IoT service providers can increase the profitability of the service by using public sensor data [8,9], rather than directly constructing sensor networks themselves. To satisfy this idea, using a mobile sink node, it is easy to collect public sensors’ data on the IoT service providers part since the public sensor network does not have a sink node, but only broadcasts its sensing data. In the case of a disaster situation, mobile nodes in the disaster area need to be changed from normal mode to emergency mode, in which nodes broadcast their data because cellular communication may be unavailable. Assume a fire has broken out in a building. The sensor and actuator networks may be destroyed in the vicinity of the fire ignition point, and so, some part of them becomes isolated from the external network, such as the Internet, even if they are still alive. Thus, they cannot send any information to firefighters.

Based on these observations, we can realize that there needs to be a system that can improvise a networking infrastructure among independent devices, systems, networks and even services in order to dynamically merge sensing and actuation infrastructure into an integrated service network and consequently enhance existing services [10,11,12] or forthcoming IoT services. To enable this concept, drones can be employed to implement such a system. The mobility and degree of freedom (DoF) of drones enables an instant deployment of networks into regions that are difficult to reach by humans or ground-based vehicles. Already, some research works [13,14,15,16,17,18] have tried to adopt the unmanned aerial vehicle (UAV) as the mobile sink node, but these research works have focused on only implementing the drones in the sensor network and have not considered the advanced operation of the drones, such as dynamic path-planning and constructing a network among the drones.

Consequently, in this paper, we propose a new platform of a UAV or drone, drone-as-a-gateway (DaaG), which can connect the sensor and the actuator networks on demand. In contrast with other research works, we have focused on ‘which sensor needs to be focused on in terms of service’, ‘which actuator has to be firstly visited by the drone to reduce the main service delay’ and ‘which data transmission method is to be used in the given environment’, namely improvement of the interoperability of the drones to connect to a separate network. We provide DaaG with the intelligence that dynamically determines the flying path for drones and to generate messages to the actuators to improve the interoperability of the DaaG. In the previous firefighting example, when DaaG is deployed in the same situation, it can directly collect sensor data, which can be used to efficiently conduct search-and-rescue and firefighting missions, and send command messages to actuators. We have implemented the proposed system with multi-rotor-based drones and then showed via simulations and experiments that DaaG can connect independent network systems and devices as a gateway and also can enable large-scale on-demand deployment of sensing and actuation infrastructure for a WSAN or IoT network as a mobile sink.

The rest of the paper is organized as follows. In Section 2, we explain the structural and functional design of DaaG. Section 3 shows the simulation results by which our system can reduce the main service delay. We present an empirical experiment and evaluate this result to verify that DaaG works well in Section 4, and finally, we conclude our paper with Section 5.

## 2. Designing DaaG

### 2.1. Design Consideration

**Figure 1:**
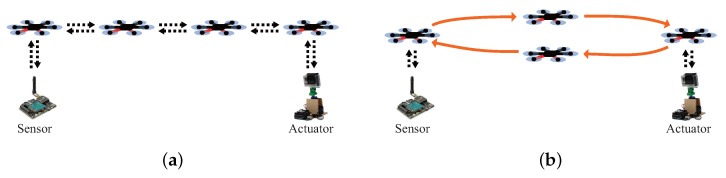
Two scenarios for the data transfer from the sensor. (**a**) multi-hop network method. (**b**) Data ferry method.

The principal features that have to consider to implement DaaG are enumerated as follows:

1. Connectivity: This enables the sensor network and actuator network to share their data when they are physically separated. Additionally, it acts as a data ferry, to deliver the data between the sensor and the actuator network, or a routing node in a multi-hop network, represented as Figure 1a, to forward the data between them. The most distinctive feature of the two methods is the mobility of the drones during the network provisioning service. In the data ferry method, represented as Figure 1b, the drones continuously travel between the sensors and the actuators. In the multi-hop network method, the drones hover and relay data between the sensors and the actuators.
2. Automated system: It can self-determine its flight plan to efficiently collect sensor data and appropriately decide the actuation.
3. Smartness: It should be smart enough to translate sensor data and actuation control between the sensor and actuator network since the latter cannot directly interpret raw sensor data that the former delivers and vice versa.

### 2.2. Structure of DaaG

**Figure 2:**
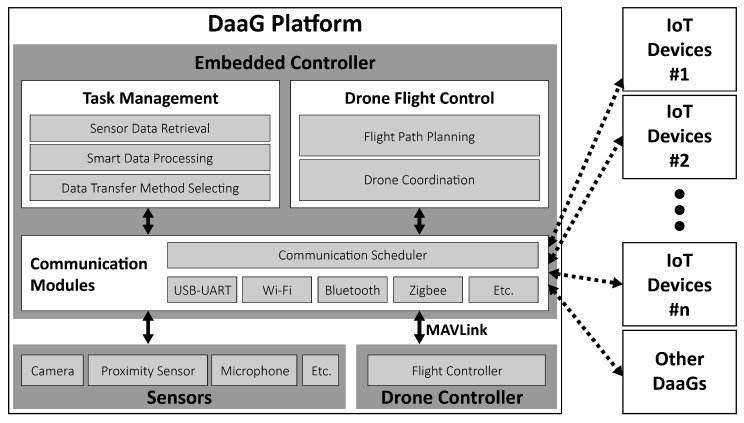
Structure of DaaG.

Figure 2 shows the structure of DaaG, which has three components: embedded controller, sensors and drone controller. The embedded controller is a core component of the DaaG platform to provide dynamic connectivity and smartness to the separate networks. The controller has three sub-components: task management, drone flight control and communication modules.

#### 2.2.1. Task Management Module

The task management module has three functionalities; sensor data retrieval, smart data processing and data transfer method selection. A sensor data retrieval function collects the data from the sensors through communication modules. These data are basically stored in the storage module. A smart data processing function uses these data in storage to generate the actuator command message. To generate the message, DaaG firstly classifies problematic sensors, which have to be monitored, and calculates the coefficient value between problematic sensors and actuators. Then, it generates the command messages for actuators to efficiently monitor problematic sensors. This component guarantees the smartness in the features. A data transfer method selecting function considers the status of the sensor network and the actuator network. Transferring data at a long range can be carried out in two ways with drones: multi-hop network and data ferry. This function selects one method that is suitable for sensing and actuating in the network environment. This component guarantees the connectivity of the features.

#### 2.2.2. Drone Flight Control Modules

There are two functionalities in the drone flight control module: flight path-planning and drone coordination. A flight path-planning function is used to plan the flight course to reduce the main service delay. The details of this function are described in Section 2.3.3. The DaaG flight maneuver requires the ability to change the path and destination during the flight, but some commercial drone controllers do not have this feature, called the off-board controller. A drone coordination function is important for the case where mission areas may overlap [19,20,21]. The module basically controls the deployment drones and prevents drone collisions, which is important in using the multi-hop network method. This components guarantees an automated system with respect to the features.

To acquire the position of the drone, the control module utilizes inertial sensors, wireless ranging sensors and/or the Global Positioning System (GPS). There are many research works utilizing these devices for positioning and navigation [22,23]. We use inertial sensors and GPS for positioning and navigation, since other methods require additional devices or infrastructure.

#### 2.2.3. Communication Modules

In the new network paradigm, sensors and actuators can have different communication interfaces. Therefore, DaaG needs to support the various communication interfaces, such as Wi-Fi, Bluetooth, ZigBee, and so on. However, since beaconing for all communication interfaces results in the overhead of the battery, a communication scheduler uses the backoff-based beaconing algorithm for energy efficiency. Moreover, DaaG supports both single-hop and multi-hop communications. Drones can communicate with each other over other drones as long as there is a connected path. This component guarantees the connectivity of the features.

### 2.3. Main Functions of DaaG

In this section, the main functions of DaaG are described in detail. The main functions are composed of communication scheduling, smart data processing, path-planning and data transfer method selection. The communication scheduling function continuously runs during DaaG operation and communicates with the sensors and the actuators. The collected data from the sensors and the actuators are utilized by other functions. The smart data processing function runs after DaaG visits every sensor node. Then, the function generates a problematic sensor list (PSL), which contains problematic sensors. The path-planning function consists of two parts: path-planning in the sensor network and path-planning in the actuator network. The former part of the function plans the flight path of DaaG and runs until DaaG collects data from every sensor. The latter part of the function determines which actuator has to be visited immediately and runs after DaaG has finished collecting sensor data. The data transfer method selection function determines which data transfer method to use. This function continuously runs during the DaaG operation. If this function decided to change the data transfer method, then all of the operating DaaGs would change the data transfer method. A more detailed description of the functions is explained in the following subsections.

**Algorithm 1** Backoff-based beaconing algorithm.
1:$\forall c_{i}\in C:c_{i,bf}=c_{i,bmax}=1$
2:**while** isDaaGWorking() **do**3:      **for each** $c_{i}$ in *C* **do**4:            $c_{i,bf}=c_{i,bf}-1$                ▹ Update remaining backoff time of $c_{i}$5:            **if** $c_{i,bf}\leq 0$ **then**6:                  $c_{i,flag}$ = runBeaconing($c_{i}$)            ▹ Broadcast beacon message with $c_{i}$7:                  **if** $c_{i,flag}>0$ **then**8:                        $c_{i,bf}=c_{i,bmax}=1$9:                        *D* = runCommunication( $c_{i}$)      ▹ Collect data *D* from nearby nodes with $c_{i}$10:                  **else**11:                       $c_{i,bf}=c_{i,bmax}=\min\left(b_{max},\alpha \;\times c_{i,bmax}\right)$      ▹ Update backoff time of $c_{i}$12:                  **end if**13:            **end if**14:      **end for**15:      **if** DaaG moves more than reset boundary length **then**16:            $\forall c_{i}\in C:c_{i,bf}=1$17:      **end if**18:      sleep($t_{period}$)                            ▹ Wait for $t_{period}$19:**end while**


#### 2.3.1. Communication Scheduling

In sensor network, sensors and actuators use sleep mode to minimize their power consumption. This technique works in the traditional environment that many sensors are stationary, but it is not appropriate in the network environment where nodes are mobile. DaaG is mobile, so the sensors’ communication interface uses sleep mode by default, and it should wake up in response to the beacon message of DaaG. Moreover, DaaG has many communication interfaces to support communicating with various sensors and actuators. There are certain advantages to reducing the energy consumption of every sensor, but DaaG consumes much energy to support beaconing for all communication interfaces.

We propose and adopt a backoff-based beaconing algorithm to reduce the energy consumption. The detailed algorithm is introduced in Algorithm 1, where *C* is the set of communication interfaces, $c_{i,bf}$ is the remaining backoff time and $c_{i,bmax}$ is the maximum backoff time of the *i*-th communication interface, $b_{max}$ is the maximum of the possible backoff time, α is the increasing backoff factor and $t_{period}$ is the time interval of the algorithm. The core idea of this algorithm is that each communication interface sends a beacon message with its inherent backoff time. Some communication interfaces increase their backoff time to reduce energy, when there are no nodes around using the same interface. Even if there are no nodes that use a certain communication interface at the current position, there is the possibility that the node appears when DaaG moves to a different position. Therefore, we adopt a maximum backoff time $b_{max}$ in the algorithm to limit the backoff increase.

The difference between $b_{max}$ and $c_{i,bmax}$ is that the former affects backoff time with respect to the movement of the drone, while the latter only considers the response of nearby nodes. However, when $b_{max}$ is too high, DaaG can still pass a node in between two beacons. DaaG also sends beacons for every arbitrary distance it moves to prevent missing nodes.

#### 2.3.2. Smart Data Processing

The smartness that differentiates DaaG from conventional mobile sinks is as follows. First, DaaG collects the data from the sensors as:

$$\langle id,pos,st,sv,timestamp\rangle ,$$

where id is the sensor’s unique ID number, pos is the position of the sensor, st is the sensor type and sv is the value of the sensor. DaaG determines that there is a problem at some area or for some objects by using the collected sensor data $sv_{t}$ and stored data $sv_{t-1}$ with the following conditions:

- the difference between $sv_{t}$ and $sv_{t-1}$ is larger than threshold value $sv_{th}$,
- $sv_{t}$ is lower than predefined lower bound $sv_{lb}$,
- $sv_{t}$ is higher than predefined upper bound $sv_{ub}$,

which can be described as follows,

(1) $$\begin{array}{ccccc} \left|sv_{t}-sv_{t-1}\right|\geq sv_{th} & \mathrm{or} & sv_{t}\leq sv_{lb} & \mathrm{or} & sv_{t}\geq sv_{ub}. \end{array}$$

Since we assumed the performance and the memory capacity of the sensor are low, we only consider $sv_{t}$ and $sv_{t-1}$. With this approach, the sensor could reduce the processing time, memory usage, and also the energy consumption. However, if the performance of the sensor is considerably higher for a complicated operation, the sensor could accumulate the data for a fixed period and analyze the accumulated value to detect the problematic sensor.

Then, id of the sensor is added in a PSL. To manage the problem, DaaG calculates the correlation value between the problematic sensor and each actuator. The correlation value between the sensor and actuator is as follows,

(2) $$corr_{s_{i},a_{j}}=\frac{\lambda }{\mathrm{dist}\left(s_{i},a_{j}\right)}+\left(1-\lambda \;\right)\frac{av_{t}-av_{t-1}}{sv_{t}-sv_{t-1}},$$

where $\mathrm{dist}\left(s_{i},a_{j}\right)$ is the distance between the *i*-th sensor $s_{i}$ and the *j*-th actuator $a_{j}$, λ is the weight value, which can be determined according to the type of service, and av is the value from the actuator. Then, DaaG finds the actuator that has the highest correlation with the problematic sensor. This means that the chosen actuator is the best to monitor the problematic sensor out of all other actuators. This sensor-actuator matching can help to solve problems and issues by making the actuator focus on the sensors. In the paper, these correlation values are used to calculate the path-planning of DaaG.

#### 2.3.3. Path Planning

Assume that DaaG knows the position of sensors and actuations, $pos_{i}=\left(x_{i},y_{i}\right)$ for ∀i∈V, where undirected graph $G=\left(V,E\right)$, *V* is a set of nodes and *E* is a set of edges. First, DaaG calculates the edge weight for all links in the *E* as follows:

(3) $$w_{ij}=\sqrt{{\left(x_{i}-x_{j}\right)}^{2}+{\left(y_{i}-y_{j}\right)}^{2}},$$

for ∀i,j∈S where S⊂V is a set of sensors.

To gather the data from the sensors, the position error of the sensor should not be larger than the communication range, in our case, about 10 m.

Then, DaaG runs a path-planning algorithm, which is described in Algorithm 2. In the path-planning algorithm, the beaconing algorithm is used to collect the sensor data. The purpose of DaaG is to retrieve the sensor data in the sensor network, which is similar to the traveling salesman problem with neighborhoods (TSPN) [24] and close enough traveling salesman problem (CETSP) [25]. Therefore, it does not need to visit all sensors in the sensor network because using wireless communication can help DaaG get the sensor data without approaching the sensor.

This point makes Algorithm 2 different from other path-planning algorithms, such as the nearest-based algorithm. However, since using this algorithm alone can result in missing a node in the sensor network, DaaG marks the flag value $F_{i}$ of the sensed node as one, and sets the flag value as two for the visited node. Finally, DaaG moves toward the node that has the lowest weight among the nodes.

**Algorithm 2** Path-planning algorithm in the sensor network.
1:**for** i=1 to |S| **do**                            ▹ Initialize all $F_{i}$ to 02:     $F_{i}=0$3:**end for**4:**while** any $F_{i}=0$ exists **do**            ▹ Run until DaaG collects data from every sensor5:     **if** DaaG visits $s_{i}$ **then**6:          $F_{i}=2$7:          $s_{k}$ = getSensorToVisit($s_{i}$)         ▹ Get unvisited sensor $s_{k}$, which has the highest $w_{ik}$8:          $\left(pos_{sx},pos_{sy}\right)$ = getPosition($s_{k}$)9:          moveDaaGTo$\left(pos_{sx},pos_{sy}\right)$      ▹ Command flight controller to move to $\left(pos_{sx},pos_{sy}\right)$10:     **end if**11:     *T* = getSensorListFromCollectedData(*D*)          ▹ Get list of sensed sensors from *D*12:     **for each** $s_{i}$ in *T* **do**13:           **if** $F_{i}=0$ **then**14:                $F_{i}=1$15:           **end if**16:     **end for**17:**end while**


Once DaaG collects all sensor data in the sensor network, it uses Algorithm 3 to move DaaG to the actuator network and to give command messages to the appropriate actuator in the case of the data ferry network method.

**Algorithm 3** Path-planning algorithm in the actuator network.
1:PSL= runSmartDataProcessing( *D*)                ▹ Get PSL with smart data processing2:VL= List(empty)                             ▹ Initialize visited list VL3:ACL = List(empty)                   ▹ Initialize actuator correlation value list ACL4:**for** j = 1 to $\left|A\right|$**do**                           ▹ Calculate $ac_{j}$ for all actuators5:     $ac_{j}=\sum_{i=1}^{\left|PSL\right|}corr_{s_{i},a_{j}}$6:     ACL.insert($ac_{j}$)7:**end for**8:**if** data ferry **then**9:     **while** VL≠A**do**                     ▹ Run until DaaG visits every actuator10:         $a_{j}$ = getActuatorToCommunicate(A\VL,ACL)       ▹ Get $a_{j}$ with highest $ac_{j}$ in A\VL11:         $\left(pos_{ax},pos_{ay}\right)=$ getPos($a_{j}$)12:         VL.insert($a_{j}$)                                ▹ Put $a_{j}$ into VL13:         moveDaaGTo$\left(pos_{ax},pos_{ay}\right)$14:     **end while**15:**else if** multi-hop **then**16:     $a_{j}$ = getActuatorToCommunicate(A,ACL)17:     $\left(pos_{ax},pos_{ay}\right)=$ getPosition($a_{j}$)18:     $s_{i}$ = getNearestSensorFrom($a_{j}$)19:     $\left(pos_{sx},pos_{sy}\right)=$ getPosition($s_{i}$)20:     **for** k = 1 to $n_{d}$ **do**21:         moveDaaGTo$\left(\begin{array}{c} \frac{k\cdot pos_{ax}+\left(n_{d}+1-k\right)\cdot pos_{sx}}{n_{d}+1},\frac{k\cdot pos_{ay}+\left(n_{d}+1-k\right)\cdot pos_{sy}}{n_{d}+1} \end{array}\right)$22:     **end for**23:**end if**


In Algorithm 3, *D* is the set of collected data, which is collected from Algorithm 1, $ac_{j}$ represents the correlation value of actuator $a_{j}$, ACL contains $ac_{j}$ values for every actuator $a_{j}$, A⊂V is a set of actuators, VL⊂A represents the set of actuators that are visited by DaaG and $n_{d}$ is the number of drones deployed. First, DaaG runs the smartness algorithm, so that it extracts a list of problematic sensors. The list has a tuple of $\langle s_{j},corr_{s_{j},a_{1}},corr_{s_{j},a_{2}},\cdots ,corr_{s_{j},a_{\left|A\right|}}\rangle$, where $s_{j}$ is the identification of the sensor and $a_{i}$ is the actuator. Finally, DaaG visits all actuators in the list in the order of the correlation value, so that command messages will be transmitted and actuators will focus on the problematic sensor. However, when DaaG selects the multi-hop network method, drones do not directly visit actuators; they only form a bridge between the sensor network and the actuator network. The sensor that is the nearest to the highest correlated actuator is selected in order to transmit the sensor data to the highest correlated actuator at first. Then, this sensor and this actuator will be connected through drones, and these nodes will act as a sink node for their network.

#### 2.3.4. Data Transfer Method Selection

To select the appropriate data transfer method, DaaG has to recognize environment variables, such as network state, service type, etc. After that, DaaG calculates a data transfer method score, which directly determines the data transfer method, as follows:

(4) $$S_{dt}=\sum_{i=1}^{n}\alpha_{i}\times \theta_{i},$$

where $\theta_{i}$ is the environment variable, such as loudness, temperature, light intensity and humidity. $\alpha_{i}$ is its weight value. For example, in the case of a wildfire detection system, the weight of temperature and light intensity would be higher than that of loudness.

Furthermore, light intensity and temperature are usually low in normal situations. In this case, the system does not need to send data continuously. Therefore, using the data ferry method to deliver the data reduces the energy consumption with no setbacks. However, if a wildfire occurs, the light intensity and temperature of the surveillance area will be increasing, and an immediate and continuous data report is required to understand the situation with accuracy. In this case, continuous data transmission with a multi-hop network is required.

The effect of environment variables could be adjusted by modifying environment weight value $\alpha_{i}$. This value could be determined by evaluating the sensors. Following the $S_{dt}$, DaaG chooses the data transfer method between data ferry and multi-hop network when $S_{dt}<Th_{S_{dt}}$. $Th_{S_{dt}}$ is the threshold value of the data transfer method score, and we calculate $Th_{S_{dt}}$ as follows:

(5) $$Th_{S_{dt}}=k\times \frac{n_{d}}{\left(n_{a}+n_{s}\right)\times dist\left(A,S\right)}$$

where $n_{d}$ is the number of drones, $n_{a}$ and $n_{s}$ are the number of actuators and the sensors, dist(A,S) is the distance between the geographical center points of the actuating area and sensing area and *k* is the coefficient to scale the $Th_{s_{dt}}$. The number of drones $n_{d}$ is proportional to the throughput of the data ferry method. However, the other values, $n_{a}$, $n_{s}$ and dist(A,S), make the travel time of the drone longer. Therefore, these values are inversely proportional to the throughput of the data ferry method. We set the value of *k* as 1600 based on our simulation result.

Furthermore, this equation can be used to optimize the number of drones to deploy. After the deployment of the sensors and the actuators, the manager of the system could collect data from the sensors and get $S_{dt}$ in the normal state. With the accumulated information of $S_{dt}$, the manager could set the threshold value $Th_{S_{dt}}$ and calculate the minimum of drones to be deployed. With this approach, we could optimize the configuration.

In the multi-hop network method, data can be continuously transferred. Therefore, it is favored when the service wants guaranteed real-time information. The communication link, however, suffers from interference from other drones. Moreover, many drones are needed to cover separate networks that are far from each other, which can cause high capital and operational expenses. This network structure can also cause a battery imbalance problem, which is a major issue in sensor networks.

In the data ferry method, the data are uploaded to the drone with maximum throughput; the drone flies to the destination, and it comes back for additional data uploading. As it is free from interferences, it encounters a much lower bit error rate. However, the total throughput of the data ferry case is related to the flying distance and speed of drones. Therefore, network efficiency may be decreased when the speed of a drone is slow or the data size is small.

## 3. Simulations

Before we implement our system on the drone, we have verified that our algorithms are suitable for connecting separate networks and can reduce the main service delay.

We have conducted a series of simulations that are designed to examine the network performance, energy efficiency, and path-planning capability of our system. The result of each simulation is given in the following subsections.

### 3.1. Network Performance

Two of the main features of our proposed system are the capability to interconnect isolated networks and to combine independent services. The performance of such features is heavily dependent on the network performance, which our proposed system tries to enhance. With the following simulations, we examine the performance of our proposed system’s network. To simulate the methods as realistically as possible, we employ D-MUNS [26], a multiple UAV network simulator, which is based on ns3 [27] and RotorS [28].

#### 3.1.1. Simulation Setup

**Figure 3:**
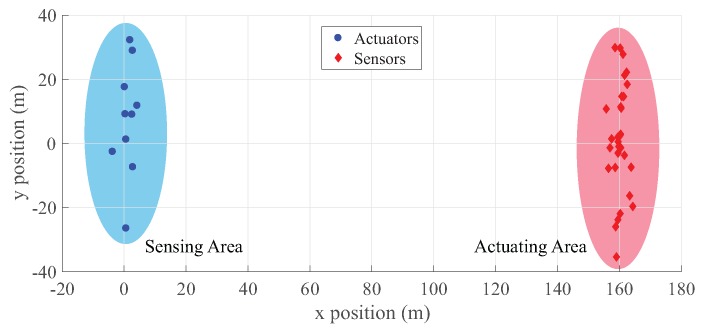
Node placement of the network performance simulation.

We have deployed sensors and actuators as in Figure 3, and more detailed simulation configurations are as follows;

- The number of sensors $n_{s}$ in the sensing area is 30.
- The number of actuators $n_{a}$ in the actuating area is 10.
- The number of drone $n_{d}$ is one.
- The distance between the sensing area and actuating area is 160 m.
- Drones going to the actuator pass the sensing area.
- The problematic sensor is the southern-most node in the sensing area.
- All sensors, actuators and drones are equipped with a Wi-Fi interface for communication.
- The maximum throughput of sensors is limited to 100 Kbps

In our simulations, we select a priority service throughput as a metric to analyze the performance of our algorithms. The priority service throughput is the sum of weighted actuators’ throughput. The weight value is the correlation coefficient of actuators regarding the problematic sensor. Therefore, the priority service throughput $Th_{ps}$ is as follows,

(6) $$Th_{ps}=\sum_{i=1}^{n_{a}}\omega_{i}\times Th_{i}.$$

The reason for adopting the new metric is that the main actuator (the highest-correlated actuator) is most important in the service, but other actuators can also monitor the problematic sensor imperfectly. In our simulations, $\omega_{i}$ is selected in accordance with the reciprocal of the distance between the problematic sensor and the actuators. The set of $\omega_{i}$ in our simulations is as follows: $\left\{0.23,0.30,0.39,0.51,0.67,0.87,1.13,1.47,1.92,2.50\right\}$.

#### 3.1.2. Simulation Results in the Data Ferry Method

We implemented two path-routing algorithms in the simulator, which are a nearest neighbor algorithm [29] and a rendezvous algorithm [30], for the comparison study, since these algorithms have been widely used for UAV path-finding. All three algorithms firstly visit the sensor network and then visit the actuator network. The nearest neighbor algorithm visits the unvisited nearest sensor node in a greedy manner and then visits the actuator network in the same way. In case of the rendezvous algorithm, eight sensors and four actuators are selected as rendezvous points. Then, the drone visits only 12 rendezvous nodes since other sensor nodes or actuator nodes are connected to rendezvous nodes. Our event-centric path-planning algorithm visits the sensor node following our algorithm, which is a little bit like the rendezvous algorithm, but it firstly visits the highest correlated actuator.

**Figure 4:**
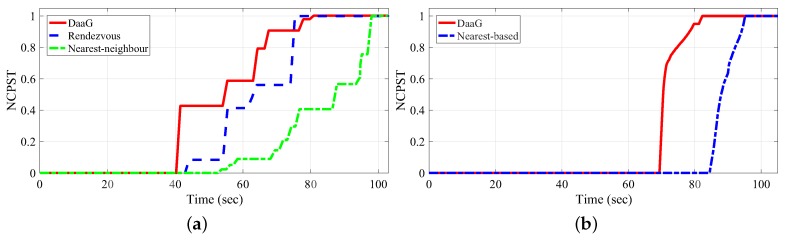
Normalized cumulative priority service throughput. (**a**) Data ferry method; (**b**) multi-hop network method.

The data transmission is not continuous in the WSAN. And this makes the network throughput comparison difficult. Therefore, to ease comparison of the performance, we adopt normalized cumulative priority service throughput (NCPST). With the NCPST, the weighted size of transmitted data could be obtained. As we can see from the result of the simulation in Figure 4a, our event-centric path-planning algorithm in DaaG shows the best performance compared to the other two algorithms in the simulation. With respect to the time it takes for the drone to send the sensing data to one of the actuators successfully, our algorithm is 6.5% earlier than the rendezvous algorithm and 23.2% earlier than the shortest-path algorithm.

The rendezvous algorithm generates circular path which connects rendezvous nodes based on the geographic position of the rendezvous nodes. After the path has created, the drone follows the generated path to collect the data and returns to the starting point. However, our event-centric path-planning algorithm generates path based on the correlation between the actuators and the sensors. This is what differentiates our algorithm from the rendezvous algorithm. Therefore, the rendezvous algorithm’s arrival time at the actuator network is delayed. In terms of the priority service throughput, that of DaaG showed the fastest growth rate. Because the first actuator that the other two algorithms visited is not a highly-correlated actuator, it has a low $\omega_{i}$ value. The rendezvous algorithm achieved the maximum throughput at first since the total service delay is lower than the other algorithms, but this is not more important than the main service delay. Hereby, we can verify that our path-planning algorithm can increase the main throughput in the data ferry method.

#### 3.1.3. Simulation Results in the DaaG Multi-Hop Network Method

In this simulation, we select a nearest-based multi-hop network as a comparison target, which organizes a multi-hop topology to connect a sensor and an actuator that has the shortest distance to the sensor. Similar to the previous simulation, drones visit the sensor network and gather the sensing data. The differences are that the number of drones is increased to four, and they create a bridge for the communication.

Since the distance between the sensor network and actuator network is too long to connect these two networks with a single drone, we use more drones to connect the networks with the multi-hop method.

Figure 4b shows the result of the simulation. Our video [31] shows the whole simulation procedure and result. In the result, our drones create a communication bridge from the highest correlated actuator to the sensor that is closest to the actuator. However, the nearest-based path-planning scheme connects a sensor and the actuator that is closest to the sensor, but not the highest correlated actuator. As shown in Figure 4b, DaaG’s communication has started 17.7% earlier than the nearest-based path-planning scheme. Furthermore, we can notice that DaaG’s priority service throughput increased more quickly than the other. Moreover, the DaaG system quickly finishes the data transmission compared to the nearest-based path-planning scheme since all actuators complete their job of monitoring the problematic sensor. This is because the highest correlated actuator is the first monitor in the actuator network, but it is the third monitor in the nearest-based path-planning scheme. Following the analysis, we can claim that DaaG can provide new services faster than other path-planning algorithms in terms of both the data ferry and multi-hop network method. Moreover, DaaG can select the message transfer schemes according to the network status and service type.

#### 3.1.4. Network Throughput Comparison between the Data Ferry and Multi-Hop Method

**Figure 5:**
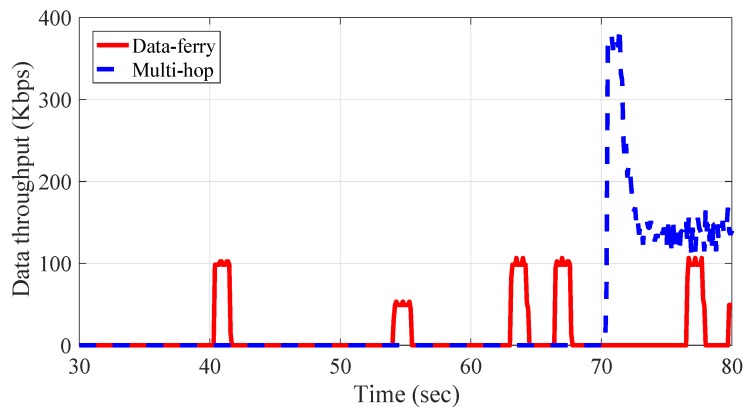
Throughput comparison between the data transfer methods.

Since DaaG selects the data transfer method for a given environment, we measured the performance of each data transfer method. The result is shown in Figure 5. When the connection is built, the throughput of the multi-hop network method is higher than the data ferry method.

The multi-hop network method could transmit data at the maximum throughput continuously. However, the data ferry method only transmits data at the maximum throughput when it communicates with nearby sensors or actuators. However, to construct a multi-hop network, drones have to fly to the points and hold their position. Therefore, the network construction time of the multi-hop method is higher than the time of the data ferry method. Furthermore, sensors and actuators have to continuously transmit/receive the data.

### 3.2. Energy Consumption

With size-constrained devices, energy efficiency is one of the most important issues. Most sensors/actuators operate on batteries, so the lifetime of the devices is limited if the energy efficiency is not handled well. In this subsection, we have simulated the energy consumption efficiency improvement of DaaG in the environment.

#### 3.2.1. Simulation Setup in the Energy Consumption Simulation

The simulation has been conducted with the OPNET simulator. Throughout the entire simulation, 36, 64 and 100 sensors were uniformly deployed in an 180 m × 180 m, 240 m × 240 m and 300 m × 300 m area, respectively. Furthermore, the manet_station model of OPNET is used as the sensor model. Each node transmits a 200-byte packet with a 45-m communication range to the sink node at one of the corners. The packet generation follows a Poisson distribution with a rate of 0.1 packets per second. The optimized link state routing (OLSR) protocol is used in the multi-hop scenario, and DaaG collects the data physically.

To measure the energy consumption of each node, we used the first order radio model [32]. In this model, the energy consumption for transmitting 1 bit over a distance *d* is $E_{Tx}=E_{elec}+\epsilon_{amp}\times d^{2}$, where $E_{elec}$ is the energy spent by the transmitter or receiver circuit and $\epsilon_{amp}$ stands for the energy dissipated in the transmit amplifier. The energy used to receive 1 bit of data is $E_{Rx}=E_{elec}$. In the series of simulations, $E_{elec}$ was set to 50 nJ/bit, and $\epsilon_{amp}$ was set to 100 pJ/bit/m${}^{2}$ [33,34]. Each simulation lasted 900 s.

#### 3.2.2. Simulation Result

**Figure 6:**
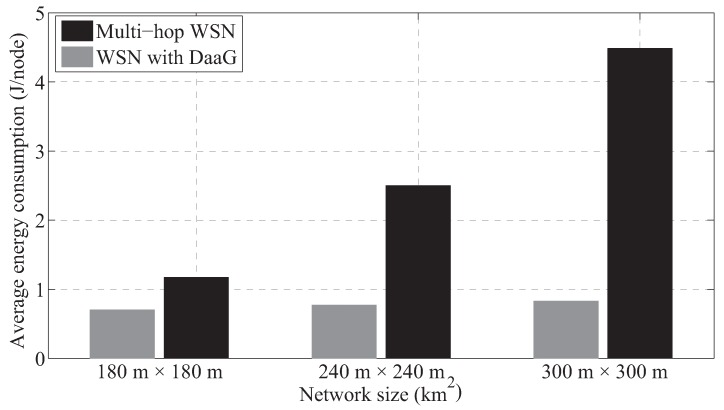
Average energy consumption with various network sizes.

Figure 6 compares the average energy consumption of the two cases: multi-hop network and DaaG deployment. With any size of network, DaaG performs much better than the multi-hop network. Sensors with DaaG deployment only need to deliver data to the DaaG in a single-hop manner, so that the energy is kept low and is much less affected by the size of the network. It is also true that the drone will consume a large amount of energy, but the energy constraint is not concerned with the total energy consumption, but the longevity of the sensors.

**Figure 7:**
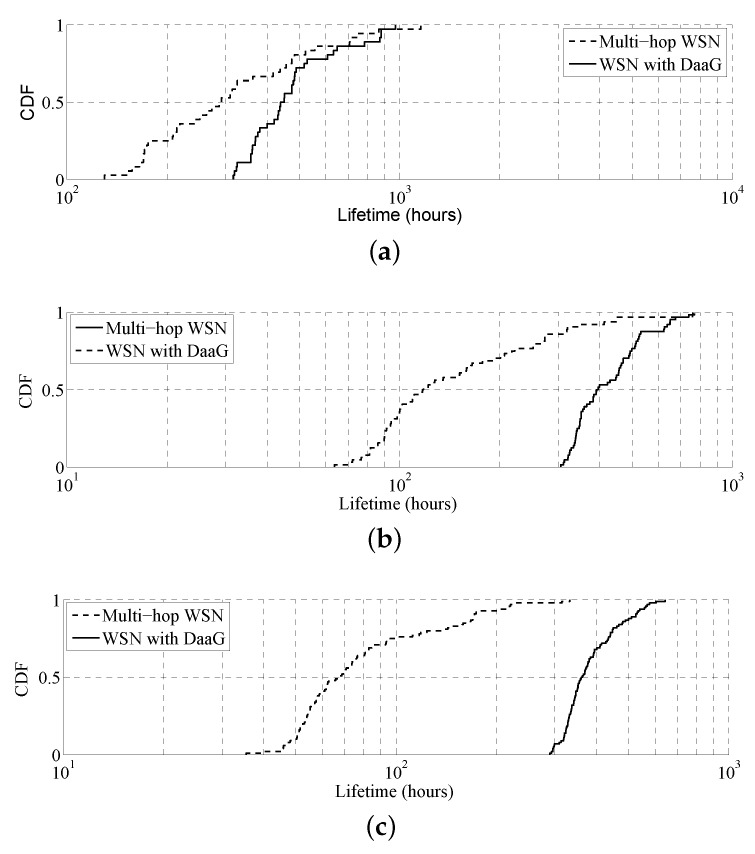
Cumulative distribution function of the lifetime under various network size conditions. (**a**) 180 m × 180 m. (**b**) 240 m × 240 m. (**c**) 300 m × 300 m.

**Figure 8:**
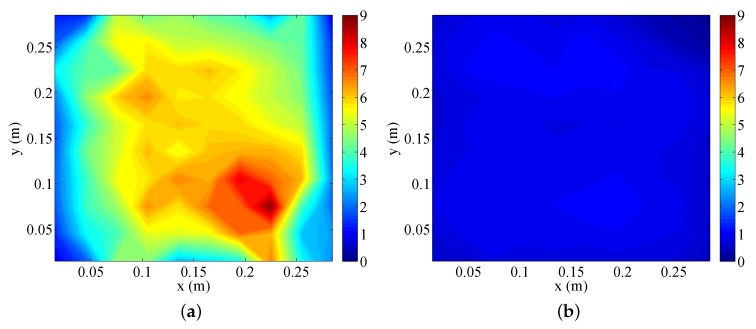
The map of sensor energy consumption. (**a**) Multi-hop network. (**b**) DaaG deployment.

Figure 7 presents the cumulative distribution function (CDF) of the lifetime for various network sizes. The lifetime of each sensor can be estimated with an arbitrary value of 1296 joules, a typical AAA battery size. As we are interested in the ratio of the two cases, not the exact time, the exact size of the battery is particularly important. As can be seen in the figure, DaaG deployment always shows a much longer lifetime than traditional multi-hop WSN. As the network size increases, the performance gap is widened. Moreover, the lifetime of the sensors varies largely in a multi-hop network, which is a crucial setback for the WSAN management. Figure 8 shows the energy consumption map of each scenario. We can observe that sensors located around the sink node consume more energy in the multi-hop network. This is because data packets are forwarded through the nodes around the sink. On the other hand, the energy consumption is balanced when DaaG is applied to the network. It can also be observed that the energy consumption over all regions of DaaG deployment is similar to the minimum energy consumption in the multi-hop network.

### 3.3. Main Focusing Delay

To validate our system, we have analyzed the delay of our system measured throughout the simulations. In the analysis, two metrics related to delay are considered: a main focusing delay and a total service delay. The main focusing delay means the interval in which a main actuator, which can best monitor problematic sensors, starts to monitor problematic sensors, after it completes collecting all sensor data and determining the problematic sensors. This delay is a core metric to determine whether the system can serve the main content rapidly. The total service delay is the elapsed time to complete visiting all actuators, which include both the main actuator and the non-main actuators, and send the operation message to each actuator, when the start time begins after all sensor data have been collected. Since the non-main actuators may monitor the problematic sensors or provide some help to achieve the requested service, the total service delay is one of the important metrics to determine whether the system can serve the requested content perfectly.

#### 3.3.1. Simulation Setup

The following simulation has a setup that is similar to that of the simulation in Section 3.1. The details are as follows. The numbers of actuators in the actuating area are 5, 10, 20, 40, 60, 80, 100, 120, 160 and 200, and the number of sensors in the sensing area is three-times that of the actuators. The distance between the sensing area and the actuating area is 160 m, and a drone covers the entire region. The drone moves passed the sensing area to the actuators. With every simulation, one or more problematic sensors are randomly selected. It takes 10 s for the drone to complete the command operation. With each setup with the same number of sensors and actuators, 100 simulations were conducted. The following is the result.

#### 3.3.2. Simulation Result

**Figure 9:**
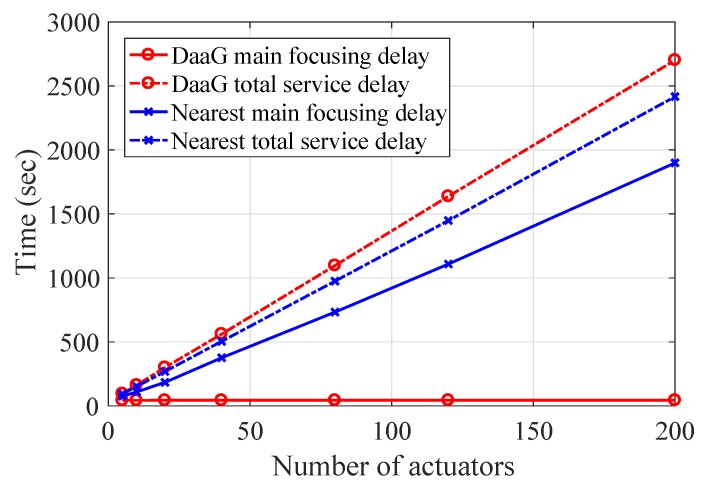
Delay measurement from the simulation.

Figure 9 shows the result of delay measurement with our simulation. The result is desirable in that the main service delay is shorter than the nearest-based method. This is mainly due to the fact that the nearest-based path-planning method is likely to visit some nodes that are not main actuators. On the other hand, DaaG visits the main actuators with higher priority with the smartness function. The time saved with the smartness function enables DaaG to outperform the nearest-based method by far, under certain conditions. Figure 9 shows that the total service delay of DaaG is longer than the nearest-based method, as DaaG considers the task priority more important than the overall time delay. Yet, DaaG spends only 13.9% longer than the nearest-based path-planning method even with 200 actuators; thus, the scalability is good.

## 4. Implementing and Evaluating DaaG

In this section, we empirically present the feasibility of DaaG based on a specific scenario where the sensing area is physically separated from the actuation area. We firstly describe a network configuration as a plausible scenario, then explain how to implement DaaG and relevant network nodes for sensors and actuators, and after that, we explain the empirical results and their analysis.

### 4.1. Exemplary Scenario

The purpose of our experiment scenario is to connect separate sensor and actuator networks.

**Figure 10:**
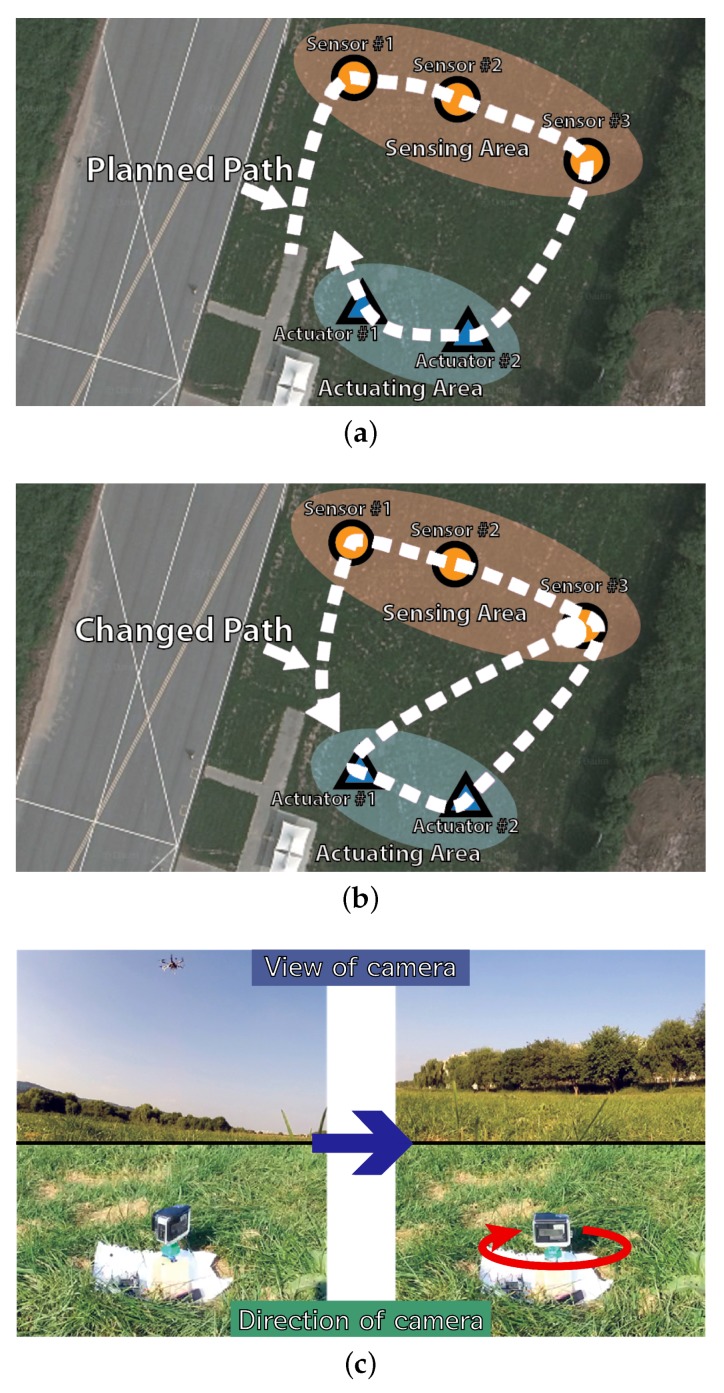
The pictures of the demonstration scenario including path change information. (**a**) Experiment setting and expected DaaG path before the experiment begins. (**b**) Path change due to the retrieved data. (**c**) Actuator changed by DaaG .

To conduct the experiment, we have deployed sensors and actuators as in Figure 10a. The whole scenario of our experiment is as follows:

1. There are two separated areas, a sensing area and an actuating area, any one of which is not connected to the other.
2. Sensors in the sensing area measure the light intensity value.
3. Actuators in the actuating area are filming the sensors, but the number of actuators is smaller than the sensors.
4. Drone #1 (DaaG) flies over the sensing area, associates itself with sensors in the sensing area and retrieves the sensed data from the sensors.
5. Drone #1 visits the actuating area and calculates the angle of the actuators.
6. Drone #1 sends the angle data to the actuators to film the sensor that detects an event.

The following implementation, deployment and evaluation will be explained based on this configuration and scenario, but the simulation study will use different network configurations and scenarios.

### 4.2. Implementation

In this subsection, the implementation details of the system are elaborated. Based on our prior work [35,36], we extended the functionality of our novel drone system to realize the proposed DaaG. As in Figure 2, the DaaG implementation is composed of two parts: software and hardware. To reflect the scenario, we implement the two separate networks: the sensor network and the actuator network.

#### 4.2.1. DaaG Hardware

**Figure 11:**
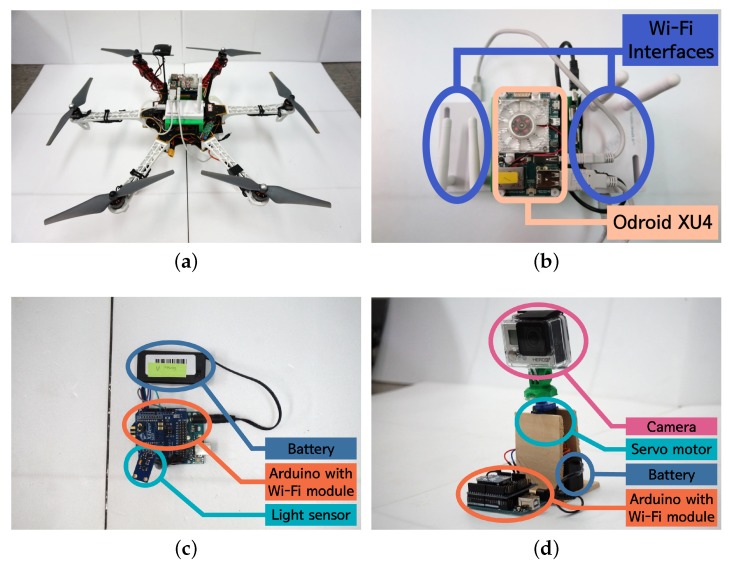
The implementation of DaaG, sensors and actuators. (**a**) The hexa-copter-based DaaG. (**b**) The computing module. (**c**) The IoT sensor module. (**d**) The IoT actuator module.

The DaaG hardware is composed of a drone frame, an embedded computer and auxiliary sensors, as in Figure 2. The pictures of the implementation are given in Figure 11a,b. We use DJI F550 Flame Wheel as a hexarotor frame. The 3DR Pixhawk flight controller [37] is installed for basic flight control, such as attitude and position control. For advanced computing operations, we chose Odroid XU4. This computing unit manages tasks, plans the flight path, sends commands to the Pixhawk and communicates with nodes or other DaaGs. The Pixhawk and Odroid are connected via a USB-UART module and communicate with each other by using MAVLink [38].

#### 4.2.2. DaaG Software

There is an Apache server running on DaaG, to handle requests from the nodes. Alongside the Apache server, MySQL DB and PHP scripts are used to enable easier manipulation of the server. Nodes in our implementation make HTTP requests to send and receive data. DaaG mimics the HTTP requests with a DTN-like communication style is needed. Normal communications are made using typical TCP communication.

In the implementation scenario, DaaG has to collect the light intensity value from the sensors, determine which area needs to be monitored and calculate the angle of the actuators to watch a certain area. Algorithm 4 shows the procedure of DaaG operation designed for the implemented scenario. In the algorithm, *S* is the set of sensors, *A* is the set of actuators, *H* is the desired heading for the actuators, *getIntensityDiff*($s_{i}$) is the function returning the intensity difference of node $s_{i}$ and *calculateActuatorDirection($a_{j},s_{i}$)* is the function that returns the heading direction of the given actuator $a_{j}$ to the sensor $s_{i}$. The process of the algorithm is as follows. The drone visits all sensor nodes. While visiting all nodes, the drone receives light condition data and generates light condition change data. After the data collection, the drone calculates the command, which makes the actuator change direction. After the commands are generated, the drone visits the actuators and rotates their cameras toward the sensor with the highest light condition change data.

**Algorithm 4** DaaG IoT control decision algorithm.
1:enableBeaconing()                       ▹ Run Algorithm 1 in background2:**while** isDaaGOperating() **do**3:     $\Delta \;lux_{max}=0$4:     $s_{max}=0$                               ▹ Initialize variables5:     collectDataFromSensors()                 ▹ Run Algorithm 2 to collect data6:     **for** $s_{i}\in S$ **do**7:            Δ$lux_{i}$ = $getIntensityDiff(s_{i})$8:            **if** $\Delta \;lux_{max}<\Delta \;lux_{i}$ **then**9:                 $\Delta \;lux_{max}=\Delta \;lux_{i}$10:                 $s_{max}=s_{i}$11:        **end if**12:     **end for**13:     **for** $a_{j}\in A$ **do**14:            $h_{j}$ = $calculateAcutatorDirection(a_{j},s_{max})$15:     **end for**16:     commandToActuators(H)             ▹ Run Algorithm 3 to control actuators17:**end while**


#### 4.2.3. Prototypes for Sensors and Actuators

There are two node types, a sensor node and an actuator node, in our experiment. Figure 11c is a sensor-mounted prototype, and Figure 11d is an actuator-mounted prototype. We use Arduino Uno for the controller and the Xbee Wi-Fi module as a network interface. On the sensor node, the BH1750FVI light sensor is attached to collect the light intensity. For the actuator node, a camera is mounted and films the environment. A servo motor is attached to the camera to adjust the viewing direction of the camera.

#### 4.2.4. Drone Control System

The DaaG ground control station (GCS) is written in Python, which could run on almost any computing hardware. The GCS manages the information of every active DaaG and commands to the DaaGs in the situations of ‘low battery’, ‘possibility of collision’ and ‘urgent change of point of interest’. To achieve the features mentioned above, the GCS receives information of DaaGs with a UDP socket and sends drone control commands with a TCP socket when needed. Since the mission duration is important for the performance of DaaG, we enabled the drone hand-off, which has been proposed in [39], to lengthen the duration.

### 4.3. Empirical Deployment

Following the experimental scenario, we have conducted the experiment in an outdoor environment. Figure 10a shows the basic layout of our experiment where the location of experiment is Seoul, Korea, where the latitude is 37.547329, and the longitude is 127.119588. The size of the experimental setting is 36.61 m by 40.14 m. The distance between sensors and actuators is about 31 m. In order to prevent sensors from communicating with actuators directly, we reduced the transmission power of the sensors’ network interface to 1 dbm. The velocity of DaaG is set 3 m/s. The threshold value for distinguishing a problematic sensor node $I_{th}$ is set to 10,000.

If the value of the light intensity sensor reaches $I_{th}$, then the DaaG system assumes that the sensor node is a problematic sensor node.

To prove that the DaaG system has smartness and works well in the separated network environment, we have chosen a nearest-based path-planning algorithm as a comparison target, which is a general algorithm for UAV that can reduce the travel time and battery consumption. With both algorithms, we conducted an empirical study to monitor sensors that have a value less than the threshold value. Furthermore, we selected the data ferry method to deliver sensing and actuating data in our outdoor experiment.

### 4.4. Empirical Evaluation

With the implemented sensors, actuators and a drone with DaaG in the actual deployment, we conducted an experiment according to the experiment scenario, observed the results and examined them.

#### 4.4.1. Observing Empirical Results

Figure 10 shows the demonstration of the actual experiment conducted. Our video [40] shows the whole experimentation procedure and result. The detailed procedure and result of the experiment are as follows:

- First, based on the starting position of Drone #1 and the location of the sensing and actuating area, the expected path of Drone #1 is in Figure 10a for both the DaaG and nearest-based planned path drone.
- Since the light intensity value of Sensor #1 is lower than the threshold in our scenario, Drone #1 runs Algorithm 3, then Sensor #1 is registered in PSL, and $corr_{s1,a1}$ will be the highest.
- After that, Drone #1 changes the path to visit Actuator #1 rather than Actuator #2, and Figure 10b shows the changed path after running Algorithm 3.
- When Drone #1 has arrived at Actuator #1, it sends an actuation message, which rotates the servo motor to focus on Sensor #1, to Actuator #1, as Figure 10c shows.

In the case of the nearest-based planned path drone, the path for traveling in the sensing area was the same as that of the DaaG and the planned path. However, the drone visits Actuator #2 first, following the planned path, which is different from the path DaaG chooses. The drone sends the order message to Actuator #2, then it goes to Actuator #1.

We can see from the experiment that the separated network infrastructure is connected immediately. Before using DaaG, the sensor network can only sense the light intensity, and the actuator network can only film some places. However, after using DaaG, these networks can act as a surveillance system for the brightness problem. This system can create a new value, which the existing network cannot provide.

#### 4.4.2. Examining Observations

As a validation, we have analyzed the delay of our system with a series of experiments. In the analyses, both the main focusing delay and the total service delay are considered, so that the observations made for the field experiment match those for the simulations. In our scenario, the main actuator can be Actuator #1, because Sensor #1 is registered in PSL and Actuator #1 is close to Sensor #1 compared to Actuator #2.

**Figure 12:**
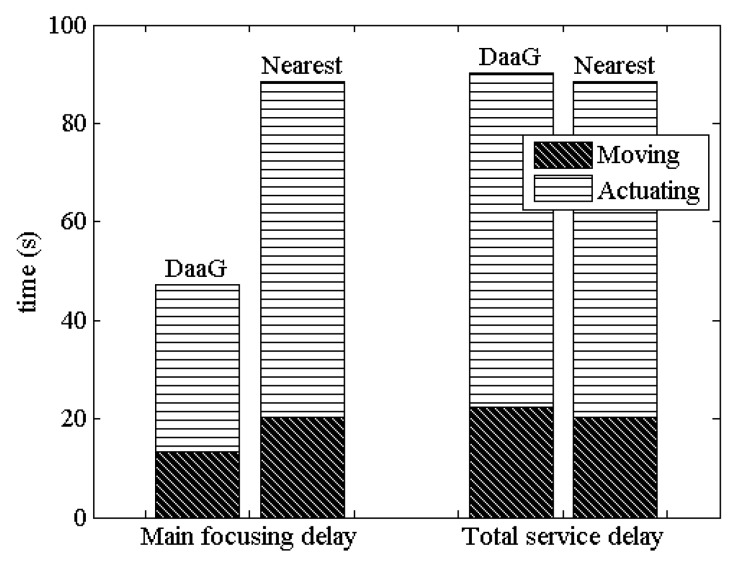
Delay measurement from the experiment.

Figure 12 shows the result of delay measurement based on our experiment. In the result, it can be seen that DaaG shows a better result compared to nearest-based path-planning with respect to the main focusing delay. The entire delay of our system for moving the drone and operating the actuator is 46.46% shorter than that of the nearest-based path-planning system. In our experiment, the nearest-based drone visited Actuator #2, which is not the main actuator. It can be seen from Figure 12 that the total service delay of DaaG is only 2.08% longer than that of the nearest-based path-planning system.

With these results, we should notice that the essential purpose of this system is to serve a new service by connecting separate networks. Following this purpose, the main focusing delay is more important than the total service delay, because a short main focusing delay can provide the priority service rapidly. The results shows that our platform is much faster than the nearest-based path-planning system, so that the main service can be rapidly provided with our platform. Naturally, the total service delay is also important to increase the quality and accuracy of the service, as a lengthy delay can negatively affect the service quality. However, our system requires only 2.08% additional time, which is negligible, compared to the nearest-based path-planning system.

## 5. Conclusions

We have designed and implemented DaaG, a drone that provides connectivity between separate networks as a mobile gateway. Our simulation and experiment results show that DaaG can connect between the sensor network and actuator network. Furthermore, DaaG uses various algorithms to connect these separate networks efficiently.

There is a large variety of possible application scenarios for the system. The drone can fly to dangerous regions that people cannot reach. Therefore, a disaster situation is the most appropriate example for this scenario. Another example is an agricultural application of an IoT system. Examples of sensing devices in this scenario would be for light, temperature and moisture. Possible actuators would be sprinklers or spraying fertilizers and pesticides. With DaaG, we believe more applications in addition to these scenarios can be realized.

The smart and path-planning algorithm in DaaG can be improved, for example to be able to run appropriately in an environment in which the system does not have knowledge of the service type, nor the map of the actuators, which is our future work.

## Abbreviations

The following abbreviations are used in this manuscript:

WSAN	Wireless sensor and actuator network
DaaG	Drone-as-a-gateway
IoT	Internet of Things
M2M	Machine-to-machine
DoF	Degree of freedom
UAV	Unmanned aerial vehicle
GPS	Global Positioning System
PSL	Problematic sensor list
ACL	Actuator correlation value list
TSPN	Traveling salesman problem with neighborhoods
CETSP	Close enough traveling salesman problem
NCPST	Normalized cumulative priority service throughput
OLSR	Optimized link state routing
CDF	Cumulative distribution function
GCS	Ground control station
